# Supplementary material for: Drug2ways: Reasoning over causal paths in biological networks for drug discovery
Source: PLoS Comput Biol. 2020 Dec 2;16(12):e1008464. doi: 10.1371/journal.pcbi.1008464 (PMC7735677; doi:10.1371/journal.pcbi.1008464)
Supplement: S1 Fig — (DOCX) [file pcbi.1008464.s001.docx]

# **S1 Figure**

## **
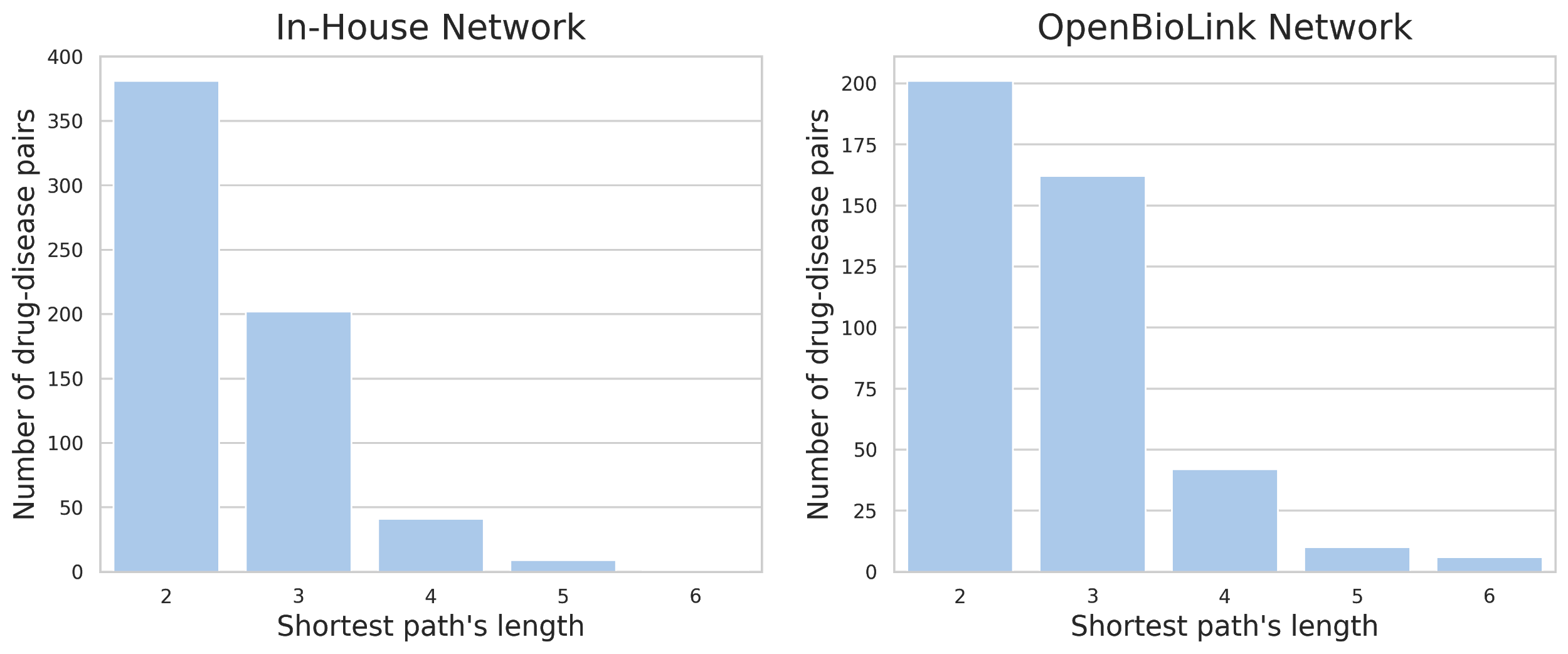
**

## **Supplementary Figure 1. Frequencies of the lengths of the shortest-paths calculated between all drug-disease pairs with *lmax <= 8* in the OpenBiolink and In-House networks.** The length of the shortest paths for the vast majority of drug-disease pairs is below 4, while a substantial number of these correspond to a direct drug-target-disease path. Interestingly, we found that the shortest paths did not include path lengths above 6. Thus, our results highlight that the shortest paths tend to be of a minimal length and potentially ignore alternative paths involving proteins that are not known disease proteins but may interact with them indirectly. Finally, for *lmax <=* 8*,* we have found that 23.599/253.638 (9.30%) and 115.640/161.040 (71.80%) drug-disease pairs possess no paths in the In-House and OpenBiolink networks, respectively.
