# Supplementary material for: Drug2ways: Reasoning over causal paths in biological networks for drug discovery
Source: PLoS Comput Biol. 2020 Dec 2;16(12):e1008464. doi: 10.1371/journal.pcbi.1008464 (PMC7735677; doi:10.1371/journal.pcbi.1008464)
Supplement: S2 Fig — (DOCX) [file pcbi.1008464.s002.docx]

# **S2 Figure**


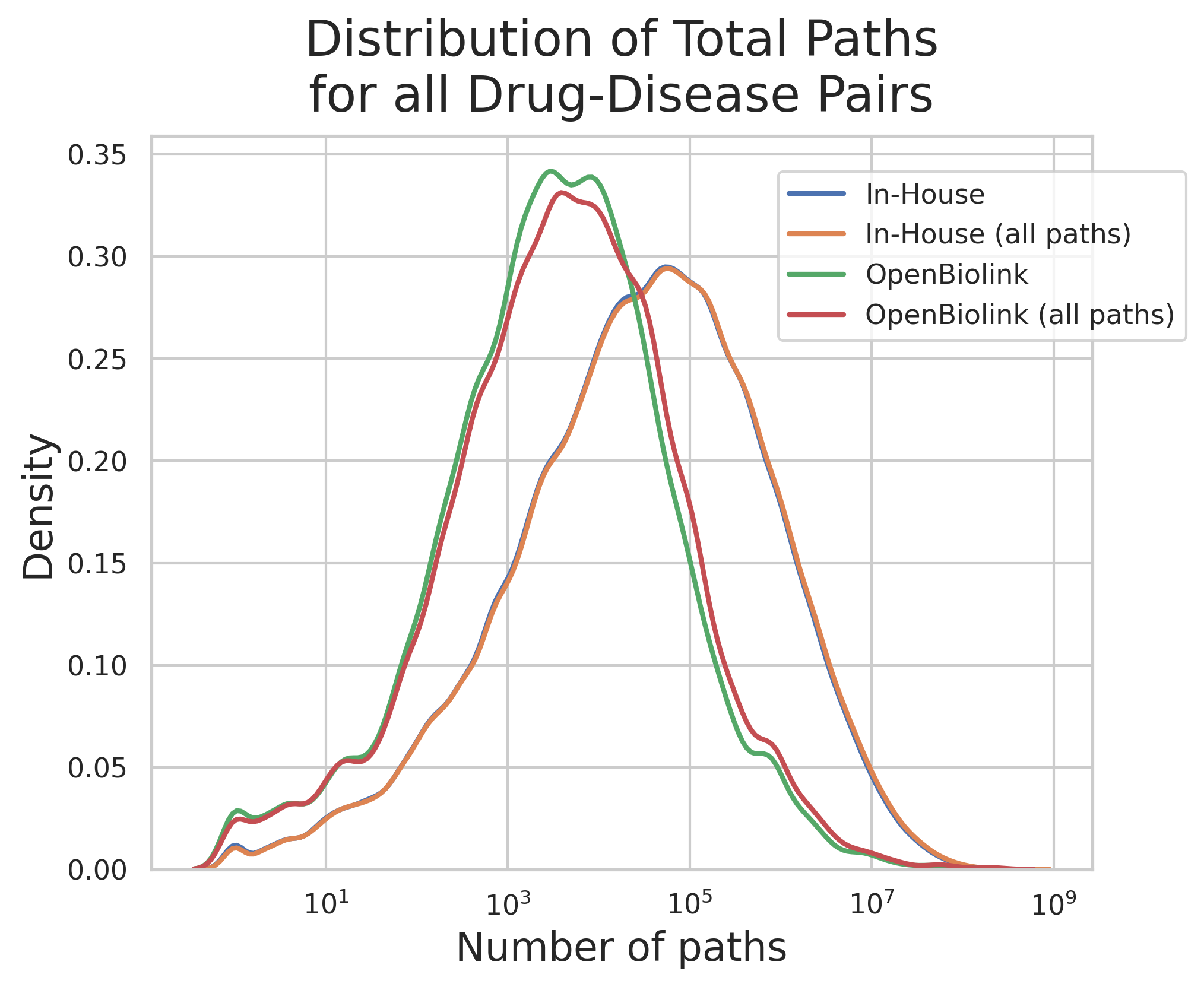


## **Supplementary Figure 2. Distribution of total paths between all drug-disease pairs in the OpenBiolink and In-House networks with *lmax =* 8.** The majority of drug-disease pairs contain a large number of paths between 1.000 and 100.000.
