# Supplementary material for: Drug2ways: Reasoning over causal paths in biological networks for drug discovery
Source: PLoS Comput Biol. 2020 Dec 2;16(12):e1008464. doi: 10.1371/journal.pcbi.1008464 (PMC7735677; doi:10.1371/journal.pcbi.1008464)
Supplement: S4 Fig — (DOCX) [file pcbi.1008464.s004.docx]

# **S4 Figure**

##


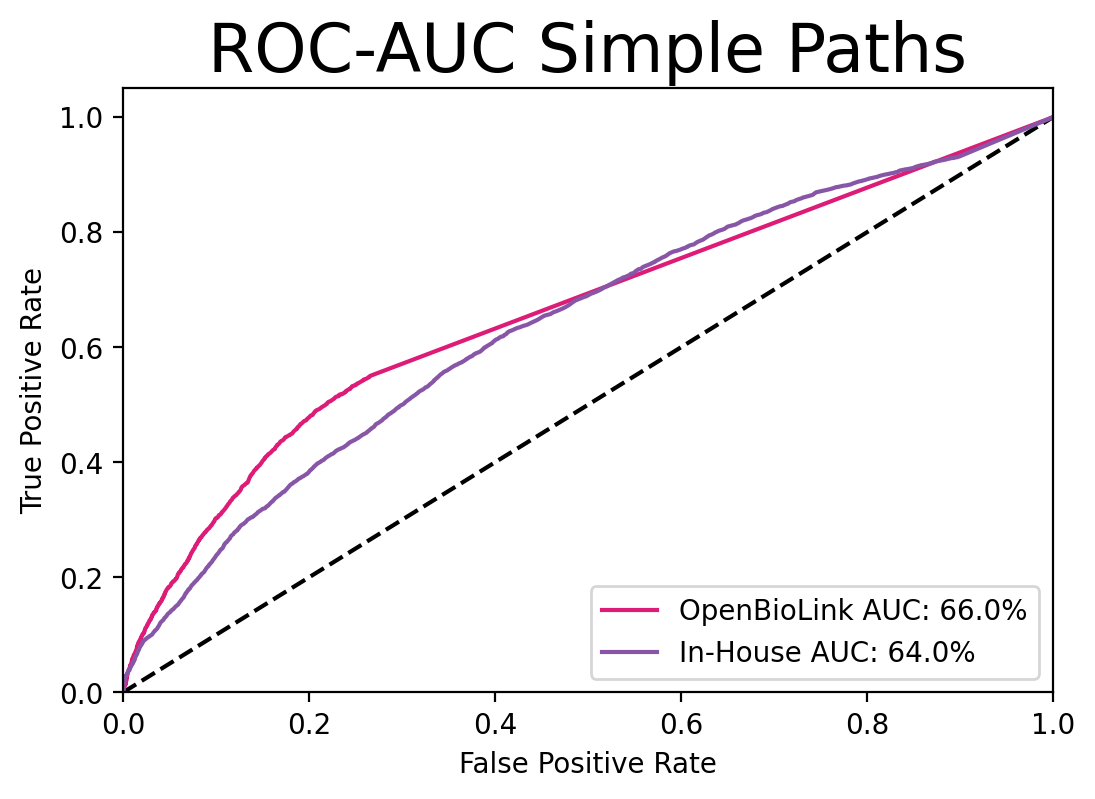


## **Supplementary Figure 4. The AUROC curves for both networks presented in the case scenario using the simple paths version of drug2ways.** The scores for each drug-disease pair are calculated by averaging the relative number of activatory/inhibitory paths between *lmax* 2 and 8 (i.e., the same *lmax* range used in the prioritization criteria). Thus, the score for each pair in each *lmax* ranges between 0 and 1, with 1 corresponding to all paths being either activatory or inhibitory, and 0 if there exist the same number of activatory and inhibitory paths). In order to include the drug-disease pairs without paths at a given *lmax* in the evaluation, these pairs are assigned a score of -1 as the score should be smaller than any drug-disease pair with paths As expected, the number of true positives predicted by drug2ways is higher at the beginning of the curve, indicating that the initial prioritized drug-disease pairs tend to be true positives relative to the non-prioritized drug-disease pairs (i.e., the ones without paths or those with an equal number of activatory and inhibitory paths). This analysis is available in the following link: <https://github.com/drug2ways/results/blob/master/validation/notebooks/auc_as_metric.ipynb>.
