# Supplementary material for: Drug2ways: Reasoning over causal paths in biological networks for drug discovery
Source: PLoS Comput Biol. 2020 Dec 2;16(12):e1008464. doi: 10.1371/journal.pcbi.1008464 (PMC7735677; doi:10.1371/journal.pcbi.1008464)
Supplement: S1 Table — (DOCX) [file pcbi.1008464.s005.docx]

# **S1 Table**

## **Inferring causal interactions**

The tables below describe the mappings between the original relationships as they appear in the networks and the two causal relationships (i.e., activation and inhibition) we subsequently infer.

| **Relation** | **Equivalent Effect (Sign)** |
| --- | --- |
| Increases | Activation (+1) |
| Regulates | Activation (+1) |
| Association (gene-disease) | Activation (+1) |
| Association (gene-phenotype) | Activation (+1) |
| Decreases | Inhibition (-1) |

**Supplementary Table 1. Relationships in the In-House network and their assigned polarity.** Mappings between original relations from source databases were made to equivalent, causal relationships (i.e., activation or inhibition). Gene-disease association edges were sourced from DisGeNet, each of which was the result of direct or indirect curation while OMIM’s list of gene-disease associations provided links to genes. Thus, the confidence of gene-disease association edges from DisGeNet is 1. Because directionality was not provided for these gene-disease association relationships, they were inferred as activation edges. Similarly, gene-phenotype association edges were sourced from OpenBioLink and were inferred as activation edges.
