# Supplementary material for: Drug2ways: Reasoning over causal paths in biological networks for drug discovery
Source: PLoS Comput Biol. 2020 Dec 2;16(12):e1008464. doi: 10.1371/journal.pcbi.1008464 (PMC7735677; doi:10.1371/journal.pcbi.1008464)
Supplement: S2 Table — (DOCX) [file pcbi.1008464.s006.docx]

# **S2 Table**

| **Relation** | **Equivalent Effect (Sign)** |
| --- | --- |
| Drug-Activation-Gene | Activation (+1) |
| Gene-Activation-Gene | Activation (+1) |
| Gene-Phenotype | Activation (+1) |
| Gene-Disease | Activation (+1) |
| Drug-Binding activity-Gene | Activation (+1) |
| Drug-Inhibition-Gene | Inhibition (-1) |
| Gene-Inhibition-Gene | Inhibition (-1) |
| Drug-Binding inhibition-Gene | Inhibition (-1) |

**Supplementary Table 2. Relationships in OpenBioLink and their assigned polarity.** While original relations in OpenBioLink were directly translated to causal relations (i.e., activation and inhibition), gene-disease and gene-phenotype associations did not contain polarity and were inferred as activation edges.
