# Supplementary material for: Drug2ways: Reasoning over causal paths in biological networks for drug discovery
Source: PLoS Comput Biol. 2020 Dec 2;16(12):e1008464. doi: 10.1371/journal.pcbi.1008464 (PMC7735677; doi:10.1371/journal.pcbi.1008464)
Supplement: S3 Table — (DOCX) [file pcbi.1008464.s007.docx]

# **S3 Table**

| **Network** | **All Paths** | | **Simple Paths** | |
| --- | --- | --- | --- | --- |
| **-** | **7/7 Activate** | **6/7 Activate** | **7/7 Activate** | **6/7 Activate** |
| OpenBioLink | 0/0 (0%) | 1/3 **(33.33%)** | 0/0 (0%) | 4/9 **(44.44%)** |
| Permuted OpenBioLink | 0/0 (0%) | 0/0 (0%) | 0/0 (0%) | 0/0 (0%) |
| In-House | 11/18 **(61.11%)** | 56/494 **(11.34%)** | 9/11 **(81.82%)** | 56/488 **(11.48%)** |
| Permuted In-House | 0/0 (0%) | 0/6 (0%) | 0/0 (0%) | 0/7 (0%) |

## **Supplementary Table 3. Results of the validation experiments focusing on prioritized drugs that activate an indication.** The table presents the validation experiments for each of the four networks (i..e, OpenBioLink, permuted OpenBioLink, In-House, and permuted In-House) using two versions of the algorithm (i.e., all paths and simple paths) based on two different criteria **(see Methods)**. For each experiment, we find a fewer number of true positive pairs prioritized in the top-ranked list that activate an indication, as compared to the number of pairs which inhibit **(Table 1)**.
