# Supplementary material for: Drug2ways: Reasoning over causal paths in biological networks for drug discovery
Source: PLoS Comput Biol. 2020 Dec 2;16(12):e1008464. doi: 10.1371/journal.pcbi.1008464 (PMC7735677; doi:10.1371/journal.pcbi.1008464)
Supplement: S4 Table — (DOCX) [file pcbi.1008464.s008.docx]

# **S4 Table**

| **Disease** | **Phenotypes** | **Source** |
| --- | --- | --- |
| Cystic fibrosis of pancreas | Chronic obstructive pulmonary disease | HPO |
|  | Exocrine pancreatic insufficiency | HPO |
|  | Elevated sweat chloride | HPO |
|  | Dehydration | HPO |
|  | Chronic lung disease | HPO |
|  | Meconium ileus | HPO |
|  | Recurrent pneumonia | HPO |

## **Supplementary Table 4. Phenotypes associated with cystic fibrosis of pancreas, the indication investigated in Subsection 2.2.**
