# Supplementary material for: Drug2ways: Reasoning over causal paths in biological networks for drug discovery
Source: PLoS Comput Biol. 2020 Dec 2;16(12):e1008464. doi: 10.1371/journal.pcbi.1008464 (PMC7735677; doi:10.1371/journal.pcbi.1008464)
Supplement: S5 Table — (DOCX) [file pcbi.1008464.s009.docx]

# **S5 Table**

| **6/7 *lmax* inhibit** | | | | |
| --- | --- | --- | --- | --- |
| **Setup** | **60%** | **75% (threshold used)** | **85%** | **100%** |
| OpenBioLink Simple | **3/18 (16.67%)** | 1/11 (9.09%) | 1/10 (10.00%) | 0/0 (%) |
| OpenBioLink All | 2/5 (40.00%) | **2/4 (50%)** | 0/0 (%) | 0/0 (%) |
| In-House Simple | **432/2397 (18.02%)** | 106/872 (12.16% | 39/308 (12.66%) | 0/0 (%) |
| In-House All | **444/2468 (17.99%)** | 105/919 (11.43%) | 36/311 (11.58%) | 0/0 (%) |

**Supplementary Table 5. Effect of the percentage of inhibitory paths on the number of true positives (6/7 *lmax* inhibit).** We apply prioritization criteria 2 and 3 while we alter criterion 1 by varying the percentage of inhibitory paths. The table presents the relative number of true positives in the list of drug-disease pairs prioritized by drug2ways for the two networks (i.e., OpenBioLink and In-House) using two variants of the algorithm (i.e., all paths and simple paths). The highest relative number of true positives for each network/variant of drug2ways is highlighted in bold.
