# Supplementary material for: Drug2ways: Reasoning over causal paths in biological networks for drug discovery
Source: PLoS Comput Biol. 2020 Dec 2;16(12):e1008464. doi: 10.1371/journal.pcbi.1008464 (PMC7735677; doi:10.1371/journal.pcbi.1008464)
Supplement: S6 Table — (DOCX) [file pcbi.1008464.s010.docx]

# **S6 Table**

| **7/7 *lmax* inhibit** | | | | |
| --- | --- | --- | --- | --- |
| **Setup** | **60%** | **75% (threshold used)** | **85%** | **100%** |
| OpenBioLink Simple | 0/0 (%) | 0/0 (%) | 0/0 (%) | 0/0 (%) |
| OpenBioLink All | 0/0 (%) | 0/0 (%) | 0/0 (%) | 0/0 (%) |
| In-House Simple | 80/191 (41.88%) | 22/54 (40.74%) | **12/22 (54.55%)** | 0/0 (%) |
| In-House All | **78/190 (41.05%)** | 20/53 (37.74%) | 12/22 (54.55%) | 0/0 (%) |

**Supplementary Table 6. Effect of the percentage of inhibitory paths on the number of true positives (7/7 *lmax* inhibit).** We apply prioritization criteria 2 and 3 while we alter criterion 1 by varying the percentage of inhibitory paths. The table presents the relative number of true positives in the list of drug-disease pairs prioritized by drug2ways for the two networks (i..e, OpenBioLink and In-House) using two variants of the algorithm (i.e., all paths and simple paths). The highest relative number of true positives for each network/variant of drug2ways is highlighted in bold.
