# Supplementary figures and images for: Drug2ways: Reasoning over causal paths in biological networks for drug discovery
Source: PLoS Comput Biol. 2020 Dec 2;16(12):e1008464. doi: 10.1371/journal.pcbi.1008464 (PMC7735677; doi:10.1371/journal.pcbi.1008464)

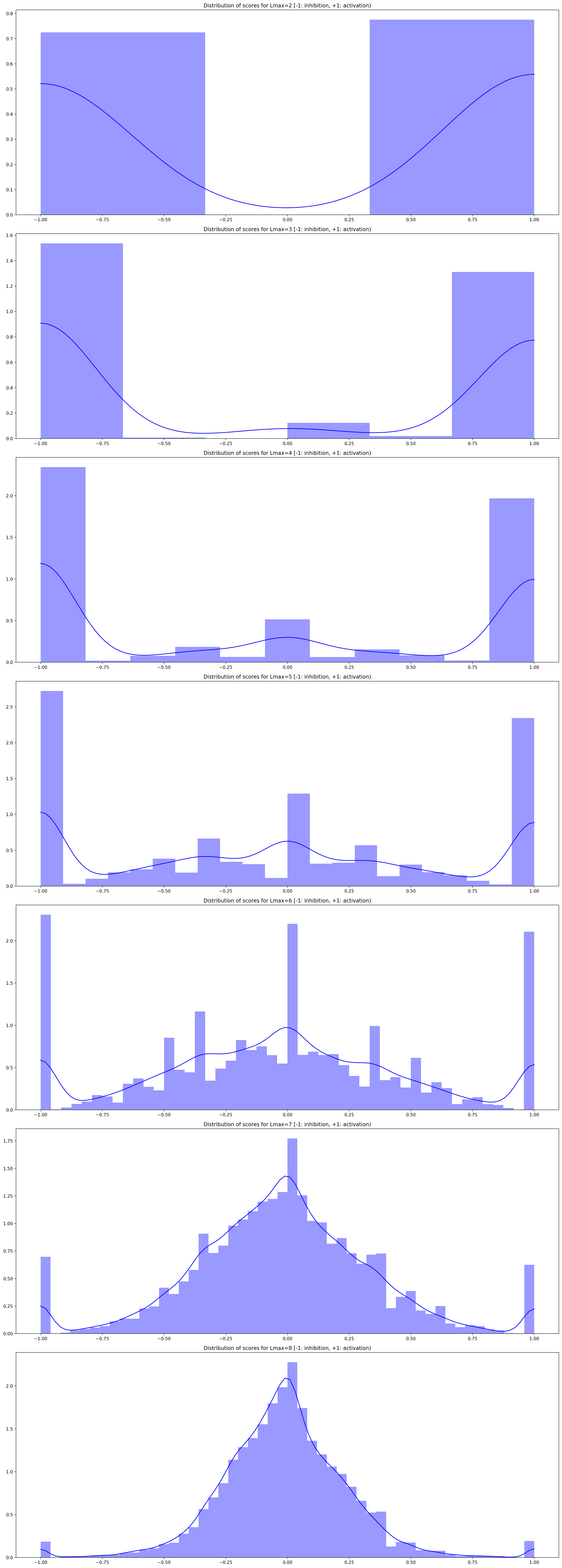

Supplement: S1 Appendix — Distribution of the scores for each lmax value on both networks (ZIP) [file pcbi.1008464.s011.zip › score_distributions/openbiolink/permuted_all_paths_openbiolink.png]

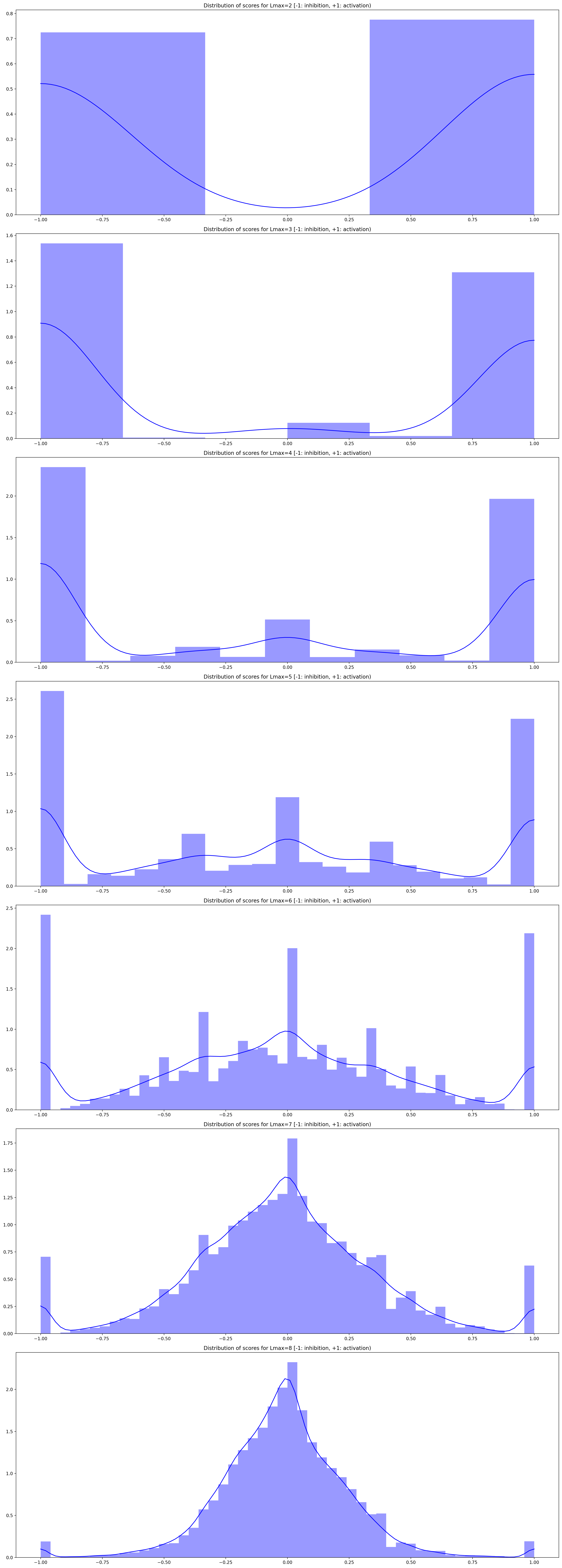

Supplement: S1 Appendix — Distribution of the scores for each lmax value on both networks (ZIP) [file pcbi.1008464.s011.zip › score_distributions/openbiolink/permuted_simple_paths_openbiolink.png]

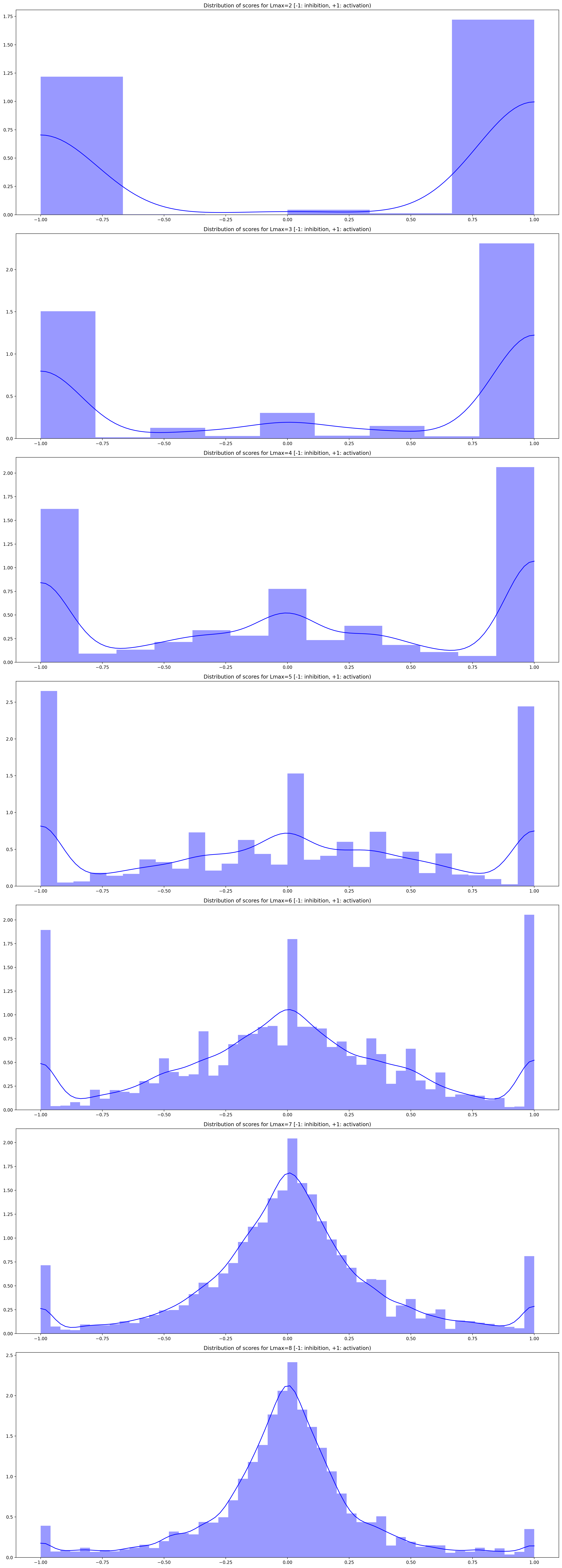

Supplement: S1 Appendix — Distribution of the scores for each lmax value on both networks (ZIP) [file pcbi.1008464.s011.zip › score_distributions/openbiolink/simple_paths_openbiolink.png]

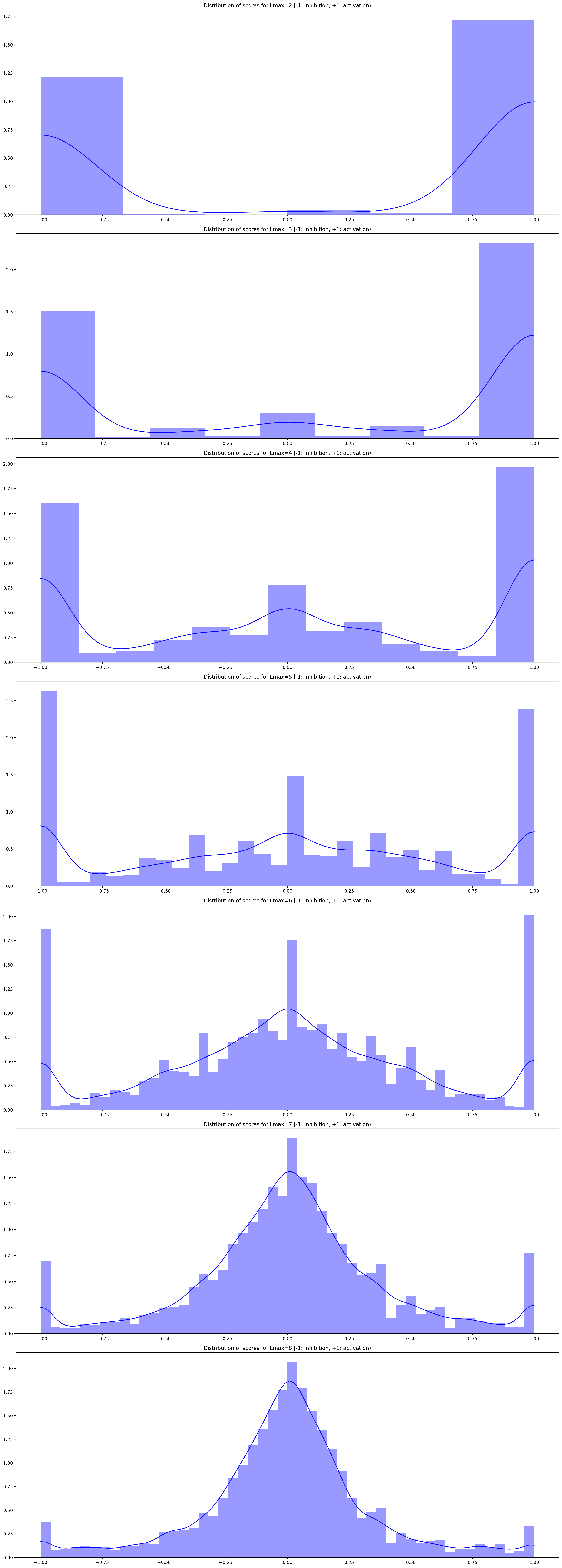

Supplement: S1 Appendix — Distribution of the scores for each lmax value on both networks (ZIP) [file pcbi.1008464.s011.zip › score_distributions/openbiolink/all_paths_openbiolink.png]

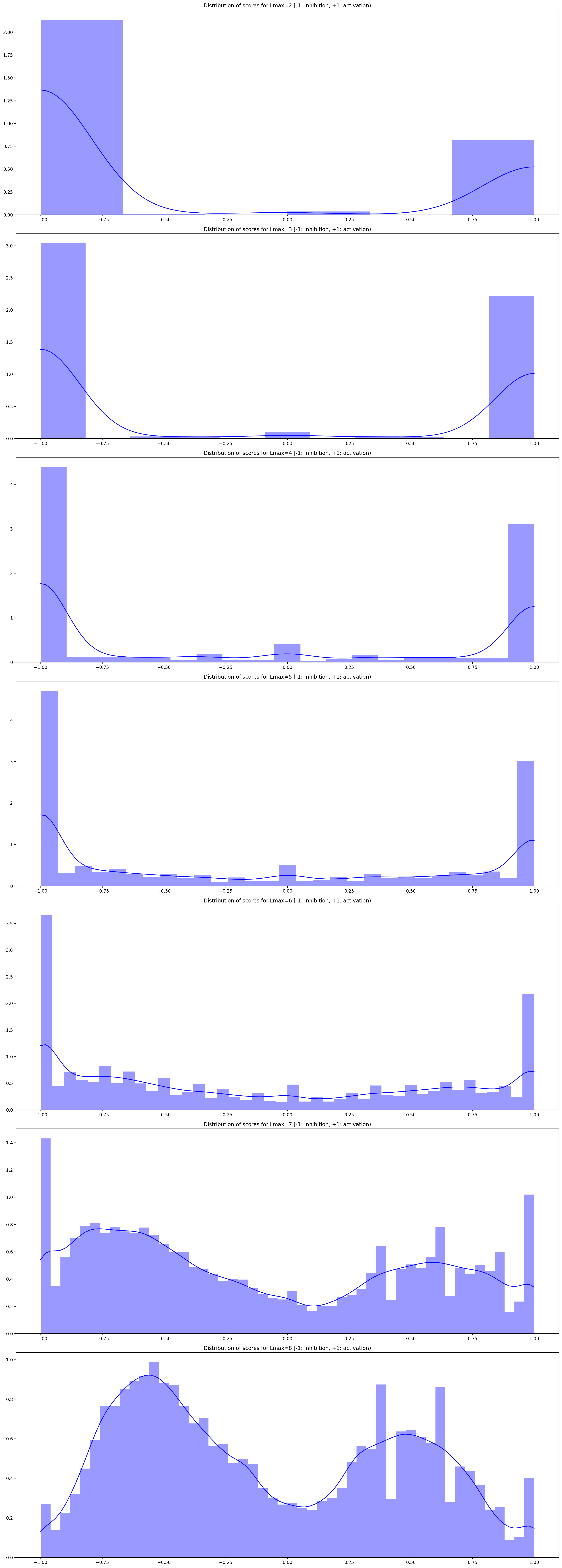

Supplement: S1 Appendix — Distribution of the scores for each lmax value on both networks (ZIP) [file pcbi.1008464.s011.zip › score_distributions/inhouse/simple_paths_inhouse.png]

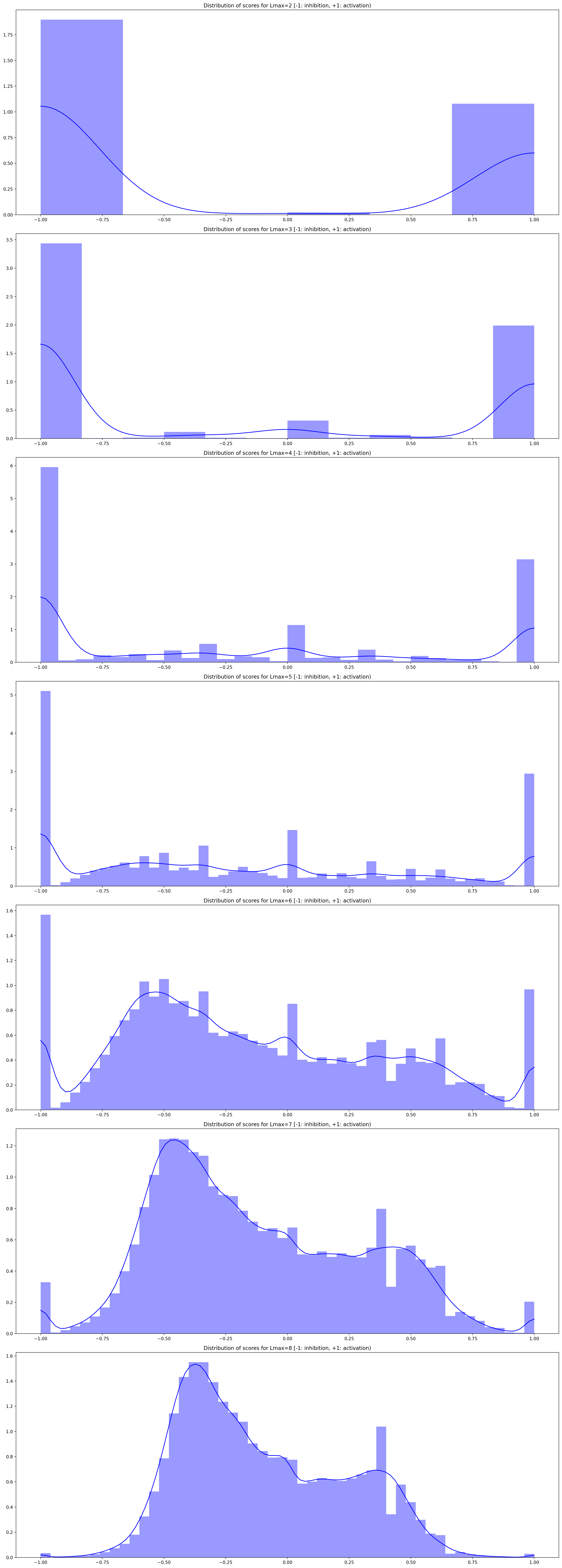

Supplement: S1 Appendix — Distribution of the scores for each lmax value on both networks (ZIP) [file pcbi.1008464.s011.zip › score_distributions/inhouse/permuted_all_paths_inhouse.png]

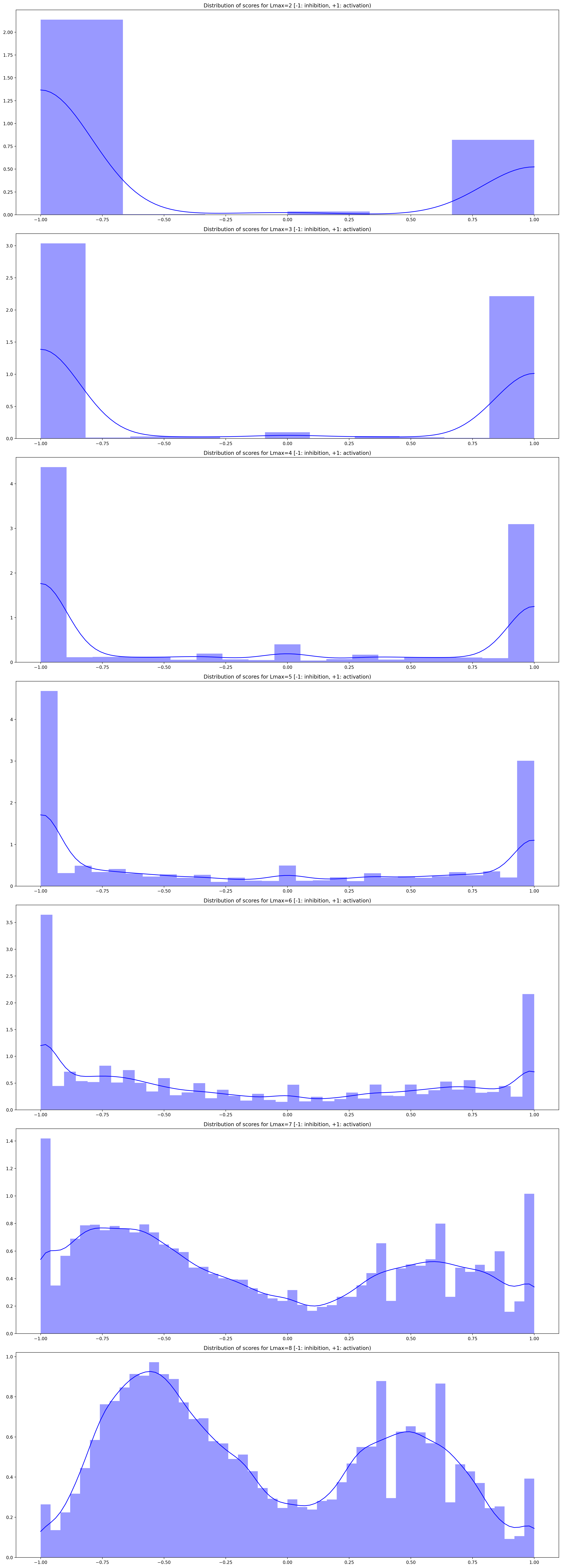

Supplement: S1 Appendix — Distribution of the scores for each lmax value on both networks (ZIP) [file pcbi.1008464.s011.zip › score_distributions/inhouse/all_paths_inhouse.png]

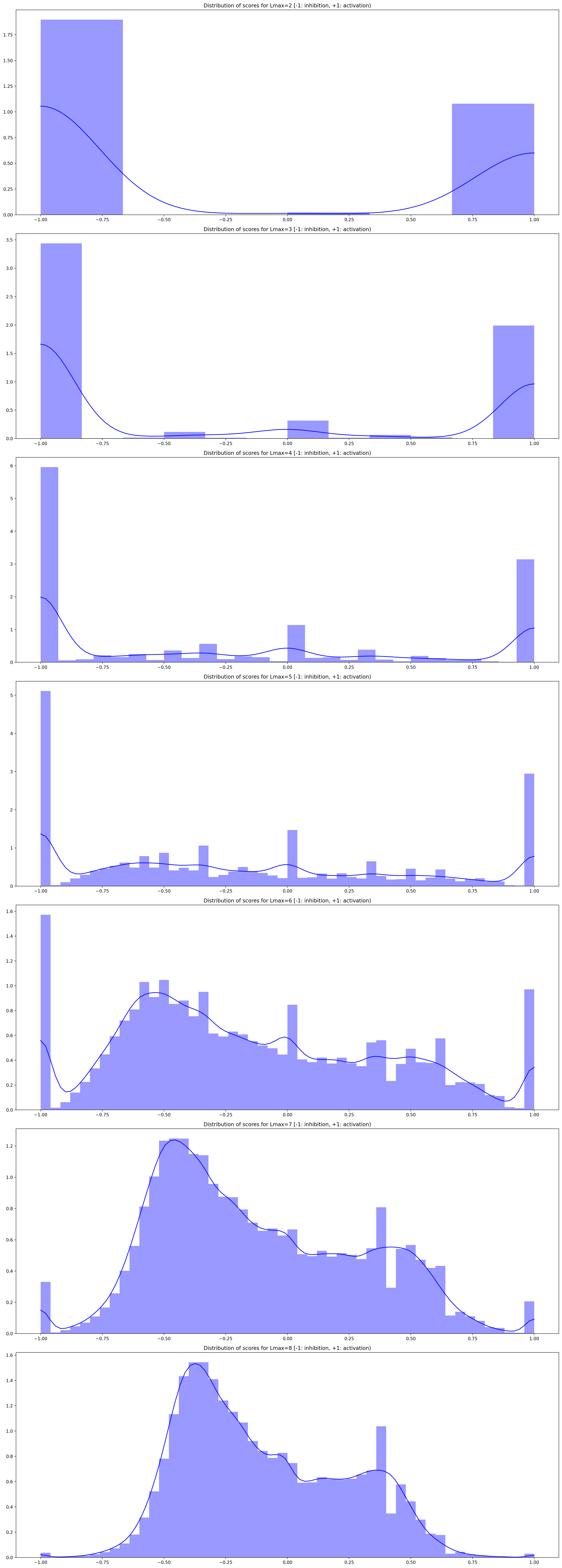

Supplement: S1 Appendix — Distribution of the scores for each lmax value on both networks (ZIP) [file pcbi.1008464.s011.zip › score_distributions/inhouse/permuted_ simple_paths_inhouse.png]
