# Supplementary material for: Drug2ways: Reasoning over causal paths in biological networks for drug discovery
Source: PLoS Comput Biol. 2020 Dec 2;16(12):e1008464. doi: 10.1371/journal.pcbi.1008464 (PMC7735677; doi:10.1371/journal.pcbi.1008464)

# **S1 Text**

## **Algorithm**

Given the problem definition, we implemented two distinct functions, namely *all_paths* and *all_simple_paths* to count activatory and inhibitory paths considering any path (i.e. including *cyclic paths*) and considering *simple paths*, respectively.

Both algorithms make use of dynamic programming and memoization to save previous solutions between a pair of nodes and retrieve them every time they are needed, as part of another problem’s solution. For *all_paths* any solution to a subproblem is enough to guarantee that we reach a valid solution to the final problem of counting paths between the source node and the target. However, in the case of *all_simple_paths*, the algorithm must be able to identify when a *cyclic path* is included in a stored solution and remove it from the total count. As partial solutions are stored as the number of accumulated paths between two nodes, there is no way to identify when a cycle may occur. One possible solution to this would be to store all paths found between two nodes and check which ones would form a cycle if added to the final solution. However, as discussed in **Subsection 4.1**, this is expensive in terms of compute time and memory, as the number of paths may grow exponentially and therefore, the space and time to store and check them does so as well. Alternatively, by keeping the list of intermediate nodes in an intermediate result, a potential cycle can be easily detected whenever a node *u* is present in the list of intermediate nodes of one of its neighbors. Then, the node must be revisited to find and remove all paths with cycles. This, however, increases the complexity of the algorithm, as a node might be revisited more than once. We analyze in detail the scalability of both versions of the problem in **Subsection 2.4**. Following, we describe two algorithms which reason over all possible paths (**Algorithm 1**) and simple paths (**Algorithm 2**) and outline their pseudocode.

**Algorithm 1** describes the pseudocode of the main function to reason over all paths from node *u* to *t* with length less than or equal to *k*. We denote paths that activate a target as activations and paths that inhibit a target as inhibitions. These activations and inhibitions are counted separately. Line 2 and 3 are the stop conditions of the recursion. Line 2 checks whether node *u* is equal to *t*, in which case, a path has been found. Line 3 checks if *k* is 0, and if so, the maximum length has been reached without finding a path to the target node and the algorithm backtracks. Line 4 initializes a counter, a pair of integers which count activations and inhibitions from *u* to *t,* respectively. Line 5 iterates over all nodes *v* in the set of neighbors of *u*. Line 6 checks whether *v* has been computed, and if so, the value is retrieved in Line 7. Otherwise, in Line 9 a recursive call is made to find all paths from *v* to *t* with a maximum length of *k - 1*. After obtaining the number of activations and inhibitions from *v* to *t*, the counter is updated accordingly in Lines 11 (if *u* activates *v*) and 13 (if *u* inhibits *v*). In Line 14, the result is saved in the cache.


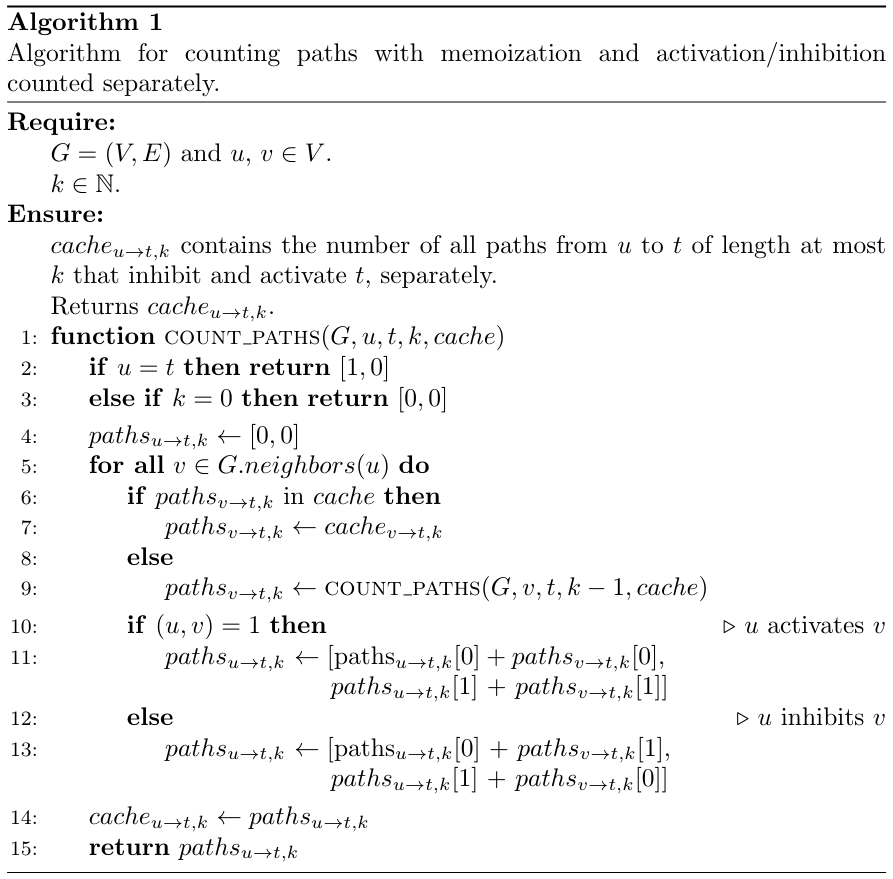


**Algorithm 2** outlines the algorithm for reasoning over all simple paths with length less than or equal to *k*. Similar to **Algorithm 1**, given any pair of nodes *u, v*$\in$*V, s.t. (u, v)*$\in$*E* and any target node *t*$\in$*P,* activations and inhibitions from *u* to *t* are recursively obtained by first calculating activations and inhibitions from any *v* to *t*. However, in contrast to **Algorithm 1**, we keep track of the list of intermediate nodes to assist with the detection of cycles. Similar to **Algorithm 1,** Line 2 and 3 are the stop conditions of the recursion. Line 2 checks whether node *u* is equal to *t*, in which case, a path has been found. Line 3 checks whether *k* is 0, in which case, we have reached the maximum length without finding a path to the target node and we need to backtrack. Line 4 initializes the counter of paths for node *u* as an empty set. Line 5 iterates over all nodes *v* in the set of neighbors of *u*. Lines 6 and 7 check whether *v* was already computed and the value is retrieved. Otherwise, a recursive call is made in Line 9 to find all paths from *v* to *t* with a maximum length of *k - 1*. After obtaining the number of paths from *v* to *t,* Line 10 determines if *u* is any of the intermediate nodes of the paths from *u* to *t*. In such a case, Line 11 revisits node *v* to get all paths from *v* to *t* that include *u* by calling the auxiliary function *get_paths_in_cycle*. Line 12 subtracts these paths from the total number of paths. Then, it is guaranteed that$paths_{v\to t, k-1}$does not count any paths including *u* and they can be added to $paths_{u\to t, k}$. This is done in Line 14, if *u* activates *v* and in Line 16 if *u* inhibits *v*. Finally, $paths_{u\to t, k}$ is stored in cache and returned as the result of the function.


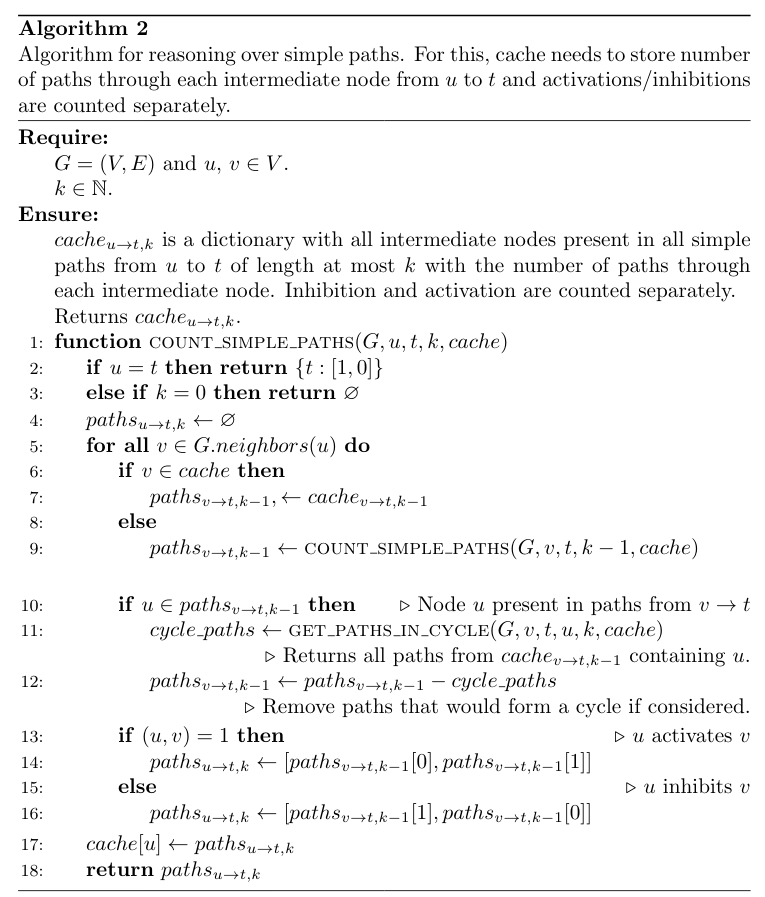

Supplement: S1 Text — (DOCX) [file pcbi.1008464.s012.docx]
