# Supplementary material for: Drug2ways: Reasoning over causal paths in biological networks for drug discovery
Source: PLoS Comput Biol. 2020 Dec 2;16(12):e1008464. doi: 10.1371/journal.pcbi.1008464 (PMC7735677; doi:10.1371/journal.pcbi.1008464)
Supplement: S2 Text — (DOCX) [file pcbi.1008464.s013.docx]

# **S3 Text**

## **Comparing distribution scores between the original and permuted networks**

To assess the robustness of the predicted scores for the drug-disease pairs investigated in **Subsection 2.1**, we compared the distribution of the predicted scores in the same range of *lmax* for each of the two networks with the score distribution from permuted versions of the original networks, generated using XSWAP (Hanhijärvi et al., 2009) and the following implementation: <https://github.com/dhimmel/xswap>. We would like to mention that this algorithm maintains the original topology and only permutes the links between edges. Thus, source nodes (drugs) and target nodes (conditions) will maintain their original topology (no incoming and outgoing edges respectively). The results discussed in this section are attached in **S1 Appendix.**

Before discussing the results, it is important to note the effect an increasing *lmax* can have on the network. Furthermore, for both versions (i.e., all paths and simple paths) a small *lmax* corresponds to a shorter and more direct path between the source (drug) and the target (disease). On the other hand, a larger *lmax* means that more paths can deviate towards distant parts of the network and the effect of feedforward loops can be captured to a greater extent by tracing various paths along them. Thus, due to this more extensive exploration, there is a point where a large *lmax* will result in paths that converge towards 0 (i.e., have an equal number of activation/inhibition interactions).

Across the two networks, we observe similar patterns in the distributions of the original versions, which contrast to those of the permuted versions, the latter of which also demonstrate marked similarities in their distribution trends. In OpenBioLink, we observe a similar pattern until *lmax*=8. This was in fact the reason why we chose this *lmax* as the upper bound for the validation. However, from this point the permuted network converges into a short-tailed distribution closely centered around 0 and the algorithm stops predicting candidate drug-disease pairs (i.e., there are no predictions close to +1 or -1). This pattern is even more pronounced for the In-House network. Here, while the original network converges and remains with its initial bimodal distribution (i.e., two consistent peaks on -1 and +1), the permuted version of the In-House network presents a short-tailed distribution that does not have any pairs in the tails of the distribution in *lmax* larger than 8. In summary, this experiment highlights that the original networks yield interesting hypotheses in contrast to the permuted networks which show no such biologically meaningful results.

**References**

1. Hanhijärvi, S., Garriga, G. C., and Puolamäki, K. (2009). Randomization techniques for graphs. In *Proceedings of the 2009 SIAM International Conference on Data Mining* (pp. 780-791). <https://doi.org/10.1137/1.9781611972795.67>
