# Supplementary material for: Drug2ways: Reasoning over causal paths in biological networks for drug discovery
Source: PLoS Comput Biol. 2020 Dec 2;16(12):e1008464. doi: 10.1371/journal.pcbi.1008464 (PMC7735677; doi:10.1371/journal.pcbi.1008464)
Supplement: Attachment — Submitted filename: Reproducible_report_PCOMPBIOL_D_20_01171.pdf [file pcbi.1008464.s014.pdf]

CENTER FOR REPRODUCIBLE BIOMEDICAL MODELING

Director: Professor Herbert M. Sauro
University of Washington, Seattle, WA
https://reproduciblebiomodels.org

Reproducibility report for: Drug2ways: Reasoning over causal paths in biological networks for drug discovery.
Submitted to: PLoS Computational Biology
Manuscript number/identifier: PCOMPBIOL-D-20-01171

Curation outcome summary: Reproduced Figure 3 and 4 using the Jupyter notebook implementation of the scripts provided.

Box 1: Criteria for repeatability and reproducibility

- [x] **Model source code provided:**
  - [x] Source code: a standard procedural language is used (e.g. MATLAB, Python, C)
    - [x] There are details/documentation on how the source code was compiled
    - [ ] There are details on how to run the code in the provided documentation
    - [ ] The initial conditions are provided for each of the simulations
    - [ ] Details for creating reported graphical results from the simulation results
  - [ ] Source code: a declarative language is used (e.g. SBML, CellML, NeuroML)
    - [ ] The algorithms used are defined or cited in previous articles
    - [ ] The algorithm parameters are defined
    - [ ] Post-processing of the results are described in sufficient detail
- [x] **Executable model provided:**
  - [x] The model is executable without source (e.g. desktop application, compiled code, online service)
    - [x] There are sufficient details to repeat the required simulation experiments
- [x] **The model is described mathematically in the article(s):**
  - [x] Equations representing the biological system
  - [ ] There are tables or lists of parameter values
  - [ ] There are tables or lists of initial conditions
  - [ ] Machine-readable tables of parameter values
  - [ ] Machine-readable tables of initial conditions
- [x] **The simulation experiments using the model are described mathematically in the article:**
  - [ ] Integration algorithms used are defined
  - [ ] Stochastic algorithms used are defined
  - [ ] Random number generator algorithms used are defined
  - [ ] Parameter fitting algorithms are defined
  - [x] The paper indicates how the algorithms yield the desired output

Box 2: Criteria for accessibility

- [x] Model/source code is available at a public repository or researcher's web site
  - [ ] Prohibitive license provided
  - [ ] Permissive license provided
  - [ ] Open-source license provided
- [ ] All initial conditions and parameters are provided
- [x] All simulation experiments are fully defined (events listed, collection times and measurements specified, algorithms provided, simulator specified, etc.)

Box 3: Rules for Credible practice of Modeling and Simulation[a]

[a]Model credibility is assessed using the Interagency Modeling and Ananlysis Group conformance rubric: https://www.imagwiki.nibib.nih.gov/content/10-simple-rules-conformance-rubric

- [x] Define context clearly: Extensive
- [x] Use appropriate data: Extensive
- [x] Evaluate within context: Extensive
- [x] List limitations explicitly: Extensive
- [x] Use version control: Extensive
- [x] Document adequately: Extensive
- [ ] Conform to standards: Insufficient

Box 4: Evaluation

- [ ] Model and its simulations could be repeated using provided declarative or procedural code
- [x] Model and its simulations could be reproduced

Summary comments: Links to the python package developed by the authors as well as scripts for generating figures were provided in manuscript (https://github.com/drug2ways/drug2ways, https://github.com/drug2ways/results.git). These were used in our attempt to reproduce the results presented in this paper. We ran the scripts in Jupyter notebooks which successfully reproduced Figure 3 and 4. Additionally, we also successfully installed the "drug2ways" python package, however no attempt to use this to simulate any results were attempted.

Anand K. Rampadarath[1], PhD
Curator
Center for Reproducible Biomedical Modeling

David P. Nickerson, PhD
Curation Service Director
Center for Reproducible Biomedical Modeling

Auckland Bioengineering Institute,
University of Auckland

[1] Email: a.rampadarath@auckland.ac.nz
