# Supplementary material for: Drug2ways: Reasoning over causal paths in biological networks for drug discovery
Source: PLoS Comput Biol. 2020 Dec 2;16(12):e1008464. doi: 10.1371/journal.pcbi.1008464 (PMC7735677; doi:10.1371/journal.pcbi.1008464)
Supplement: Attachment — Submitted filename: Rebuttal Drug2ways.pdf [file pcbi.1008464.s015.pdf]

PLOS Computational Biology

Bonn, 12. September 2020

Dear Dr. Faeder and Dr. Papin,

Please find enclosed our revised manuscript PCOMPBIOL-D-20-01171 entitled "Drug2ways: Reasoning over causal paths in biological networks for drug discovery" for publication in the journal “PLOS Computational Biology”.

We would like to thank the reviewers for their evaluation of our manuscript. We realized that our manuscript showed some shortcomings that we now erased by restructuring and modifying the text and adding a comparative study to the manuscript. We believe that we addressed all comments. Please find our point-by-point response to the reviewer comments below.

In response to the comments/questions, we have highlighted the changes within the manuscript to make our responses easy to find.

We very much hope that we addressed all issues raised about our manuscript and that it is now acceptable for publication. Thank you for your kind attention.

Sincerely yours,

Daniel Domingo-Fernández

Reviewer's Responses to Questions

Comments to the Authors:

Reviewer #1: Rivas-Barragan et al. present durg2ways, a method to calculate the proportion of pathways that possibly yield activation (or inhibition) among all possible pathways of length of at most k. The approach enumerates all paths with and without passing from cycles (refered as simple vs all paths, respectively) and combines the effect of genes on a path simply by multiplying the signs (+1 for activation, -1 for inhibition) of the pairwise relationship. The authors apply the proposed method on assessing the recovery of drug (and drug combination)-disease pairs in clinical trials.

*** We would like to thank you for your very constructive and thorough review. After addressing your concerns, we are confident that our manuscript has improved. We have addressed your comments below preceded by an asterisk symbol (*).**

The article is clearly written and aims to address an important problem in systems biology. That being said, in its current form it fails to offer sound evidence that support the validity of the claims in terms of the usability of the method for drug discovery. Accordingly, the text reads as a technical description of the algorithm without providing any biological insights. In particular:

1. The computational framework for reasoning causality over the paths of the network makes a very strong assumption that rarely hold in a cellular context: The regulatory / functional relationships between genes are assumed to be equally weighted, failing to account for the context-specificity (across different tissues, biological processes and individuals) of such kind of interactions. The authors could have a look at Vinayagam et al 2014 Nat Meth pmid: 24240319 for a signed PPI. It is also common practice to use gene (co-)expression as a proxy for weights between pairs of nodes (see Fakhry et al 2016 BMC Bioinf pmid: 27553489).

*** We share your assertion that reasoning causality over paths in a network is a strong assumption to make. However, we believe that our approach still offers biological insights, reflected in its ability to capture a greater number of existing drug-disease pairs than at random and a baseline shortest-path based approach (see answer to point 3).**

*** Firstly, to better represent the quantitative aspects of the true biology, our approach uses causal networks which can yield more information than undirected PPI networks. We have considered applying our approach on the signed PPI network presented by Vinayagam et al (2014), but unfortunately, the network was constructed based on Drosophila information and they report that only 16% of the interactions are conserved in humans.**

*** Secondly, in order to emulate how a drug may modulate a disease, we consider not only the types of interactions in our causal networks, but also the number of paths between each drug and disease. By accounting for the fact that many possible paths**

can exist between a given drug-disease pair and their cumulative effects may exert greater influence than any one path, we address a limitation of existing methods that do not take this information into account.

*** Finally, we concede that our approach currently suffers from a lack of information on the weights of each of the interactions, a limitation we have mentioned in the discussion.**

2. All of the case studies lack a proper (cross-) validation in regard to how well the method is able to predict drug (combination)-disease pairs. It would be important to define clearly the training data set (e.g., from clinical trials or previous work that uses drug indications such as Gottlieb et al 2011 Mol Sys Bio pmid:21654673, Guney et al 2016 Nat Comm pmid: 26831545) and use standardized metrics. I understand that due to the unbalance in the data and existence of possible repurposable drug-disease pairs (that are considered false positives in current training sets) AUROC might not be the best metric, nevertheless along with AUPRC (area under precision-recall curves) they do still provide a fair picture of the predictive performance of the method across different thresholds (e.g., proportion of activating/inhibiting paths to all paths).

*** Regarding the training data set (i.e., from clinical trials), we have now explained the reason for its selection in Section 4.5.2.**

*** We still maintain that reporting AUC values is not a good metric for our particular application as we narrow down the number of drug-disease pairs to a small number and we have to deal with the early retrieval problem (Berrar et al., 2012). However, we understood our argumentation was not clear in the text. Accordingly, we have now added the following text in Section 4.5.2 of the manuscript for clarity:**

"For example, imagine a scenario in which 160.000 combinations of drug-disease pairs are possible in the OpenBioLink network, and of these, 5.000 are true positives (i.e., ~3%). From all possible combinations, if we consider the top 100 pairs prioritized by drug2ways and of these, 50 (i.e., 50%) are true positives, then drug2ways has captured a significantly greater number of true positives (50%) than what is expected by chance (~3%). However, depending on the ranking of these pairs, it is possible to obtain a low AUC-ROC if the true positives are fairly distributed across this list of 100 pairs. Furthermore, some of these true positives may represent potential drug-disease pairs that have not been investigated before."

3. In relation to my previous comment, though the authors compare the method to the enumeration of paths using two graph analysis libraries, given the biological problem the tool tries to address, a comparison to the tools that aim to predict the drug-disease pairs needs to be added. In fact some of the algorithms in GUILDify suite (Guney et al 2012 Plos One pmid: 23028459, Aguirre-Plans et al 2019 JCB pmid: 30851278) do simulate paths of length at most k, similar to the proposed method. Other methods exploring the use of alternative paths have also proposed (see Shahreza et al 2017 Brief in Bionf pmid: 28334136).

*** This is a valid point. We checked your tool, GUILDify v2.0, but were unable to simulate all experiments programmatically; This would require running an experiment on the web application for each pair (disease-protein target). Furthermore, we cannot**

upload the same networks we have used in our analysis so we believe the comparison would be unfair. Thus, we decided to compare our method using all paths vs. using shortest paths.

4. Similarly, if I understand correctly from Table1 (section 2.1), the performance of the method is poor, almost as good as random selection (50%). In my opinion this case study should be clarified substantially. It is also unclear what was the top-ranked number of drug-disease pairs used in this analysis.

*** We have now clarified this point in the results and clearly outlined that random selection would be much lower than 50% (see sentence below). Regarding the top-ranked number of drug-disease pairs, is presented in Table 1. We also modified the explanation of the prioritization criteria used to obtain the top-ranked number of drug-disease pairs in 4.5.2 (i.e., three bullet points in this section and Table 5 with an illustrative example).**

-> “In comparison, the proportion of true positives with respect to all possible combinations of drug-disease pairs from ClinicalTrials.gov is 3.19% for OpenBioLink (5.151/161.040) and 3.76% (9.537/253.638) for the In-House network, highlighting the significance of our results. These proportions are equivalent to the probability of randomly picking a true positive, which is comparatively much lower than the results yielded by drug2ways..”

5. Section 2.2 lacks negative controls, that is for how many of the remaining drugs (among all the drugs tested in clinical trials) possess paths to the targets.

*** Thank you for pointing this out. This information can be inferred from Figure 2 (heatmap) and in Section 2.2, we previously stated that:**

"The heatmap shows that in selecting larger values of Imax, the vast majority of drugs (i.e., 626/671 drugs in the In-House network also in ClinicalTrials.gov) possess paths to each of the targets.”

However, this is an important consideration as it confirms that the predictions by drug2ways are not driven by the existence/absence of paths but instead by the directionality inferred through the majority of paths. Thus, we added the same information to 2.1 with a more in-depth analysis (Supplementary Figure 2).

6. Section 2.3 should also be revised to explain how exactly these combination treatments are selected and what is the significance of finding these combinations among all the possible combination treatments.

*** We have updated Section 2.3 to reflect these suggestions. While in 2.1 we focused on the validation of drug2ways, the purpose of this particular application (2.3) was to demonstrate drug2ways’ ability to efficiently propose potential drug combinations. While we have highlighted drug combinations available in the literature, we have also implemented a pathway analysis method in drug2ways to perform enrichment on the genes in a particular path to better understand the mechanism of action (https://github.com/drug2ways/drug2ways/blob/master/src/drug2ways/pathway.py).**

Reviewer #2: Reproducible report has been uploaded as an attachment.

*** We would like to thank you for your time in assessing the reproducibility of our work. As there were no issues and the reviewer has successfully installed and run the software/figures, we have not made any changes with respect to the reproducibility.**

Reviewer #3: Authors introduce a network-based approach for drug repurposing in diseases. Mainly, they leverage all possible paths and simple paths in a network composed of drug-protein, protein-protein and protein-indication/phenotype interactions where the source is drugs and targets are indication/phenotype. Although the subject and the problem that authors approach are very important, there are some shortcomings and major points that should be addressed. These points are listed below:

*** We would like to thank you for your time in reviewing the manuscript. We have addressed all of your comments below preceded by an asterisk symbol (*).**

- The details of the method and the prediction approach for drug candidates are very hard to follow in the manuscript. For example, authors state in Figure 1 that “In the example, we want to investigate whether one of the three drugs depicted inhibits an indication and its two phenotypes. While all three drugs target the disease, two of the three (i.e., drug A and C) fail to produce the desired effects (i.e., inhibition of the indication of interest and its two associated phenotypes). However, drug2ways predicts that drug B could be a promising candidate as the majority of the paths between the drug and three target nodes of interest (i.e., indication and its phenotypes) would result in their inhibition, thus producing a therapeutic effect.” However, how they determine if the relation between protein and indication or phenotype is inhibitory or activating, is missing or lost in the text. In the methods part, authors describe the theoretical background, algorithm and its complexity to find paths, but the description of the validation dataset, the method to calculate “Normalized scores of the relative effects of drugs”, parameter tuning approach are missing from the main text.

*** Thank you for pointing this out. We believe we have addressed this issue by restructuring the manuscript organization and modifying the mentioned parts (i.e., description of the validation dataset, “normalized scores”, and criteria for prioritization).**

- In the performance evaluation on page 6, “OpenBioLink showed good results (i.e., ~50% and ~10% recovery rate for all paths and simple paths, respectively)” is written. But, what is the definition of “good”?

*** Thank you for the helpful suggestion. We agree that we should have explicitly stated the comparison with random chance to define what is good. We have made this point now in section 2.1 (see below).**

-> “In comparison, the proportion of true positives with respect to all possible combinations of drug-disease pairs from ClinicalTrials.gov is 3.19% for OpenBioLink (5.151/161.040) and 3.76% (9.537/253.638) for the In-House network, highlighting the significance of our results.

These proportions are equivalent to the probability of randomly picking a true positive, which is comparatively much lower than the results yielded by drug2ways..”

- Performance comparison of the method is between the permuted networks vs original networks. An additional comparison is only based on running time of other path calculation methods. However, I can not assess from the manuscript if this drug2ways perform better that other available methods in the literature that target predicting drug candidates. A comparative performance evaluation with other available equivalent methods is necessary.

*** We have now compared drug2ways against an approach using shortest-paths (Section 2.1) since shortest-path based methods represent many of the current state-of-the-art network-based approaches (see introduction paragraph 5).**

- I do not think the filtering criteria is described in subsection 4.5.2. What is the purpose of this filtering? As in the whole manuscript, this type of critical information and their discussion is very limited.

*** We would like to thank the reviewer for pointing out this issue and we apologize for the confusion. The criteria was described in subsection 4.5.2, but was not referred to as “filtering criteria”. Instead it was simply mentioned as criteria (see Table 5 and the three bullet points in the section). To clarify this, we have now changed “filtering criteria” to “prioritization criteria” and referenced Table 5.**

- In Figure 1, an example having a cycle and the associated simple paths are given. However, visiting a node multiple times is not so feasible in biological systems. This can be added as a constraint to the method.

*** We agree that visiting a node multiple times may not be feasible nor relevant in biological systems. However, filtering cycles out in a cyclic graph increases the computational complexity of the algorithm, as described in the Supplementary Text, and makes the execution time to rapidly explode, as shown in Figure 3. For this reason, our method constrains a path to some possible maximum length (for example, lmax=7) and has the option to only include simple paths, or paths in which nodes are visited at most once (see the two variants of drug2ways: all_simple_paths and all_paths). While this was explained in Section 4.1 of the Methods section, we can see why this was not so clear. We have also added Table 3 to the manuscript with key definitions for clarity. We believe this should be clear in the current version of the manuscript.**

In general, the current state of the manuscript is not reader-friendly. There are so many parts that actually needs to go to the results part and as explained above the method details are missing. The design and flow of the manuscript needs to be rigorously reviewed and the lacking details should be completed.

*** We believe that after restructuring the manuscript, this point should be thoroughly addressed. The results should be clearly delineated in their corresponding section and missing points in the methods should now be addressed.**

Minor points:

Figure 2b. Column names in the heatmap are not properly aligned.

*** We have now fixed this issue.**

Page 13 typo "…. by means of dynamic programming and memoization ."

*** We are uncertain about the issue with this sentence. The term "memoization" here refers to a CS optimization method: https://en.wikipedia.org/wiki/Memoization (one of the authors that was not familiarized with the term also pointed out the same issue).**
