# Supplementary material for: Drug2ways: Reasoning over causal paths in biological networks for drug discovery
Source: PLoS Comput Biol. 2020 Dec 2;16(12):e1008464. doi: 10.1371/journal.pcbi.1008464 (PMC7735677; doi:10.1371/journal.pcbi.1008464)
Supplement: Attachment — Submitted filename: response.pdf [file pcbi.1008464.s017.pdf]

PLOS Computational Biology

Bonn, 18. October 2020

Dear Dr. Faeder and Dr. Papin,

Please find enclosed our revised manuscript PCOMPBIOL-D-20-01171-R1 entitled "Drug2ways: Reasoning over causal paths in biological networks for drug discovery" for publication in the journal “PLOS Computational Biology”.

We would like to thank the reviewers for their evaluation of our manuscript. We believe that we addressed all comments. Please find our point-by-point response to the reviewer comments below.

In response to the comments/questions, we have highlighted the latest changes within the manuscript to make our responses easy to find.

We very much hope that we addressed all issues raised about our manuscript and that it is now acceptable for publication. Thank you for your time.

Sincerely yours,

Daniel Domingo-Fernández

Reviewer's Responses to Questions

Reviewer #1: I thank authors for addressing most of the points I had raised earlier, improving the manuscript substantially. That being said, in relation to some of the comments I had made in the previous version, the added value the method brings has to be clarified further in my opinion:

*** We would like to thank you for reviewing our manuscript. We have addressed your comments below preceded by an asterisk symbol (*).**

1. I understand that the information on signed / directed interactions are scarce in human, yet recent studies have increased the coverage of these interactions and have shown that they are useful in the characterization of drugs and diseases (Vinayagam et al. 2016 PNAS, doi: 10.1073/pnas.1603992113; Silverbush and Sharan 2019 Nat Commun doi: 10.1038/s41467-019-10887-6).

*** From our understanding, the PPI network by Vinayagam et al (2016) is not a signed network as they only report the confidence of the edge direction but not the specific causal effect of one protein on another, for instance, whether the effect is an activation or inhibition (see screenshot below from the PPI file available at https://www.flyrnai.org/DirectedPPI/Download.jsp). The second network by Silverbush and Sharan 2019 comprises BioGRID (a PPI network), DrugBank (drug-target edges) and DCDB (information about drug combinations). Both BioGRID and DrugBank are part of one of the two networks presented in the case scenario (i.e., In-house network). Thus, running drug2ways in the network of Silverbush and Sharan would yield similar results as their network is a subset of the one presented. The remaining resource, DCDB, is unfortunately down (http://www.cls.zju.edu.cn/dcdb/main.psp) and contains information about drug combinations, which could only serve as a validation for our methodology, but cannot be integrated into a network.**

Input-node Gene Symbol	Input-node GeneID	Output-node Gene Symbol	Output-node GeneID	Edge direction score
C1orf103	55791	MNAT1	4331	0.984
MAST2	23139	DYNLL1	8655	0.743
RAB22A	57403	APPL2	55198	0.558
TRAP1	10131	EXT2	2132	0.951
STAT2	6773	COPS4	51138	0.743

Screenshot from https://www.flyrnai.org/DirectedPPI/directed_human_ppi_file.xls

2. I agree with the authors that all classifier evaluation metrics have their strengths and limitations. To understand the prediction capacity of a model, one should not rely on only a single metric such as AUROC which is rather conservative when positive data is significantly less than negative data. Nevertheless, it still provides a framework to compare different parameters / classifiers and allows standardization of performance evaluation of methods in the literature (Lever et al. 2016 Nat Methods, doi: 10.1038/nmeth.3945). Like I had mentioned before, it is common practice to use ROC curve (as a whole or partially to only

the bottom left part to address early retrieval problem as mentioned) or the area under Precision-Recall curve (AUPRC) in the case of data imbalance (Saito and Rehmsmeier 2015 PLOS ONE, doi:10.1371/journal.pone.0118432).

*** We have now evaluated the performance of drug2ways using AUC-ROC as a metric on the predictions for all drug-disease pairs present in both networks. We also clarified the shortcomings of this validation in the methods section as we believe that, given the characteristics of our setup, it reduces the evaluation to a single number that is based on all drug-disease pairs (while we are exclusively interested in the top-ranked list). The analysis is available at the following link https://github.com/drug2ways/results/blob/master/validation/notebooks/auc_as_metric.ipynb.**

3. The authors could check GUILD, the standalone version that can be used to run programmatically for any given network and input set of nodes (Guney and Oliva 2012 PLOS ONEdoi: 10.1371/journal.pone.0043557).

*** We have investigated the network algorithms present in GUILD (see below). They can be divided into two types: network-based and network-topology based prioritization methods. The first type (i.e. Functional Flow, Nabieva et al. 2005) uses annotation scores to weight the importance of the nodes. This is information we do not have in drug2ways. Methods in the second type assume all paths are equally relevant and base their scoring system on topology information, such as node degree. However, as far as we understood, all these methods were proposed for and tested on gene disease prioritization. Drug2ways, on the other hand, was proposed as a method for drug-disease pairs prioritization. Therefore, we understand that drug2ways cannot be directly compared to the aforementioned methods. Nevertheless, we feel it is an interesting line of research that we plan to follow in the near future.**

- **3.1 NetScore**. “NetScore adopts a message-passing scheme [...]. At the end of the repetition cycle, the node scores are updated according to messages received”
- **3.2 NetZcore.** “NetZcore assigns a normalized score using the distribution of the scores of neighbouring nodes”
- **3.3 NetShort**. “NetShort accumulates the weighted shortest path lengths between a node and the rest of nodes in the network, where each edge-weight is inversely proportional to the average of the scores of the two nodes connected by the edge (i.e.edges connecting high scoring nodes are shorter). [...] however note that algorithm uses the phenotypic association scores in the edge scores file [...]”
- **3.4 fFlow “**In fFlow (based on the algorithm of Functional Flow in Nabieva et al. 2005), at each iteration, **annotation scores** flow from nodes with higher score towards nodes with lower scores at the amount of the capacity of the edge through which the nodes are connected.”

  The idea for drug2ways originated from a discussion on flow network algorithms. However, in order for flow network algorithms to find the optimal flow they require not only capacities but also the flow/weights that each node adds.

Authors of fFlow make use of information from the graph we assume is unknown. From Nabieva et al. 2005:

To determine these values, we separate all experimental sources of physical interaction data into several groups, placing each high-throughput dataset into a separate group [...]. For each group of experiments, we compute what fraction of its interactions **connect proteins with a known shared function**. We assume that the reliabilities of different sources are independent, and thus conclude by estimating the reliability of an interaction to be the noisy-or of the unreliability of the underlying data sources.

- **3.5 NetRank** "NetRank (based on the ToppGene algorithm proposed by Chen et al. 2009) uses Page Rank"
- **3.6 NetWalk**
  NetWalk (based on the Random walk with restarts algorithm proposed by Kohler, et al., 2008) iteratively simulates random transitions of a walker from a node to a randomly selected neighbour node and where at any time step the walk can be restarted depending on a predefined probability. Random walk with restarts is slightly different than PageRank with priors in the way that it normalizes the link weights.
- **3.7 NetProp**
  NetProp (based on the Network propagation algorithm proposed by Vanunu, et al., 2010) modifies random walk with restarts such that the link weight is normalized not only by number of outgoing edges but also by number of incoming edges.
- **3.8 NetCombo**
  NetCombo combines the output scores from NetScore, NetZcore and NetShort in a consensus scheme by averaging normalized scores (z-scores) of a node in all of these methods. It requires the output files of NetScore, NetZcore and NetShort.

4. (Minor) The formulas in the methods section do not display properly (at least using the PDF viewer I have used).

*** Thank you for pointing this out. This is a mistake made by the automatic export from Word to PDF. As we cannot fix it in the manuscript file as we are forced to upload a word document, we uploaded the corrected formulas as a PDF in the file with the highlighted changes.**

Reviewer #3: The revised manuscript is significantly improved in terms of its flow and with the additional results and explanations. I have only two major concerns remained that needs to be addressed.

*** We would like to thank you for your time in reviewing the manuscript. We have addressed all of your comments below preceded by an asterisk symbol (*).**

1. My first comment is about the novelty of the method. Authors describe drug2ways as "a novel methodology that leverages multimodal causal networks for predicting drug

candidates”. I think that they need to explain the aspects of novelty at least in the Discussion parts in a detail, because there are multiple works using simple paths or shortest paths or all simple paths from target to source in exploring the impact of drugs at network level. In the manuscript there are some traces that authors mention about the novelty but it must be discussed in more details about the advantages of the method in a focused way. The limitations and future additions of the method are solidly described in the Discussion and I think the novelty aspect should be also discussed extensively by referencing to the current literature in the Discussion.

*** Thank you for the suggestion. We have made this point now in the discussion.**

2. My second comment is about the tuning of the parameters. As I understood from the manuscript, the parameters of the model are the path length (k) and the percentage of inhibitory paths. Authors test multiple k values and nicely details its output in prediction, however the percentage of inhibitory paths value is not tuned anywhere in the manuscript. The value is constant as 75%. How is that value determined? If that would be tuned in an interval how the performance of the method would change? Or, if they have already done this and determined 75% accordingly, I would like to see the performance evaluation at least in the Supplementary Material.

*** We would like to thank the reviewer for bringing this point. We have now included the requested analysis in Supplementary Tables 5 and 6. Our findings show that the optimal value for the relative inhibition for these two particular networks is between 60% and 75%. Given our already restrictive criteria, higher relative inhibition values (80%-100%) yield almost no prioritized drug-disease pairs.**

A minor point is that mathematical symbols in the equations are missing in the Methods part of the revised manuscript.

*** Thank you for pointing this out. This is a mistake made by the automatic export from Word to PDF. As we cannot fix it in the manuscript file as we are forced to upload a word document, we uploaded the corrected formulas as a PDF in the file with the highlighted changes.**
